# Supplementary figures and images for: A versatile site-directed gene trap strategy to manipulate gene activity and control gene expression in Caenorhabditis elegans
Source: PLoS Genet. 2025 Jan 22;21(1):e1011541. doi: 10.1371/journal.pgen.1011541 (PMC11753634; doi:10.1371/journal.pgen.1011541)

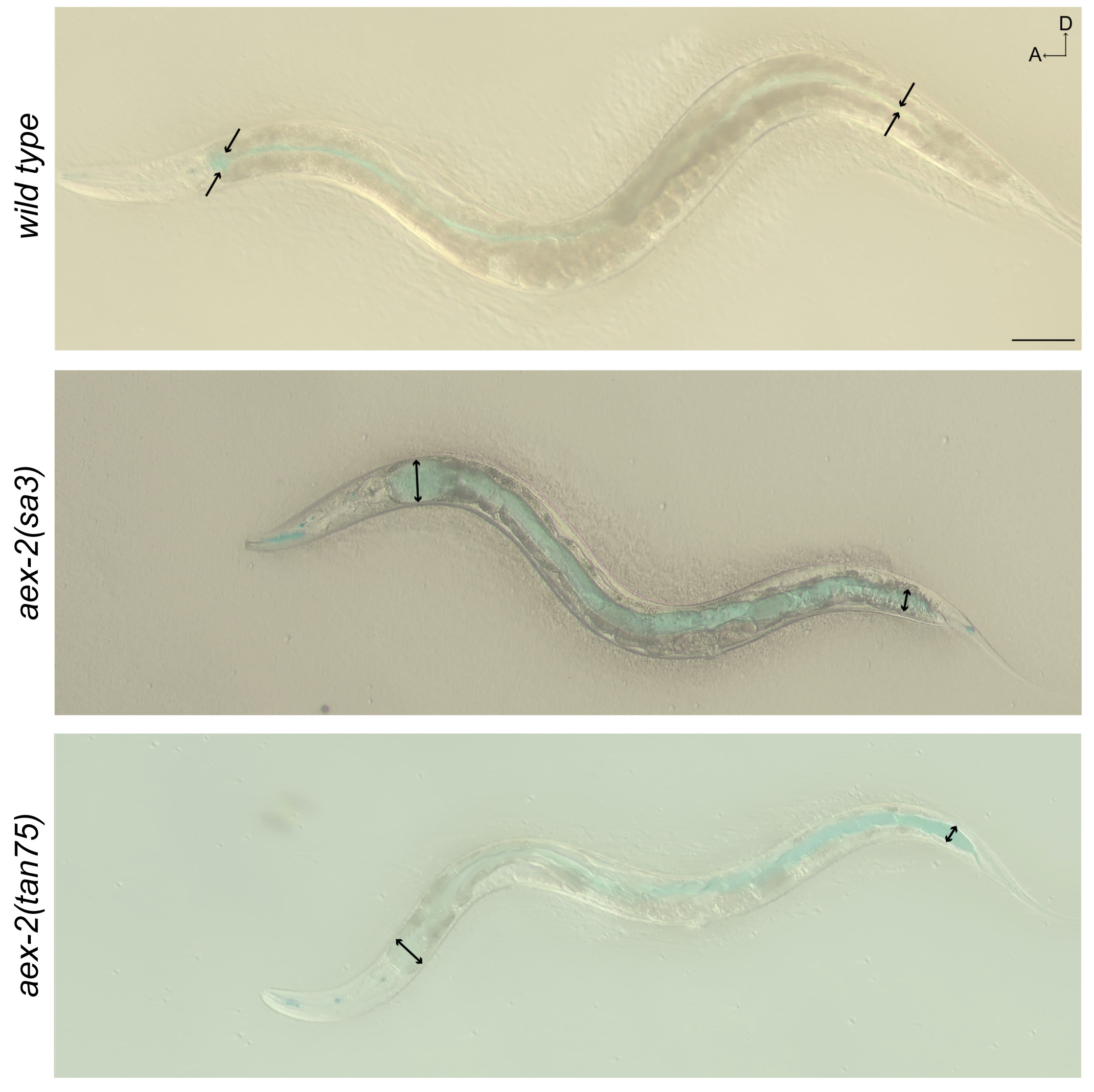

Supplement: S2 Fig — Intestinal lumen was visualized by feeding adult worms with bacteria OP50 in blue food dye. For wild-type animals, the intestinal lumen of wild-type worms is thin; for aex-2(sa3) and aex-2(tan75[cGAL GT]) mutants, the intestinal lumen is severely distended, particularly in the anterior and posterior sections of the intestine. Black arrows show an increased distention of the intestinal lumen of aex-2 mutants compared to wild-type worms. Scale bar is 50 μm. A, anterior; D, dorsal. (TIF) [file pgen.1011541.s002.tif]

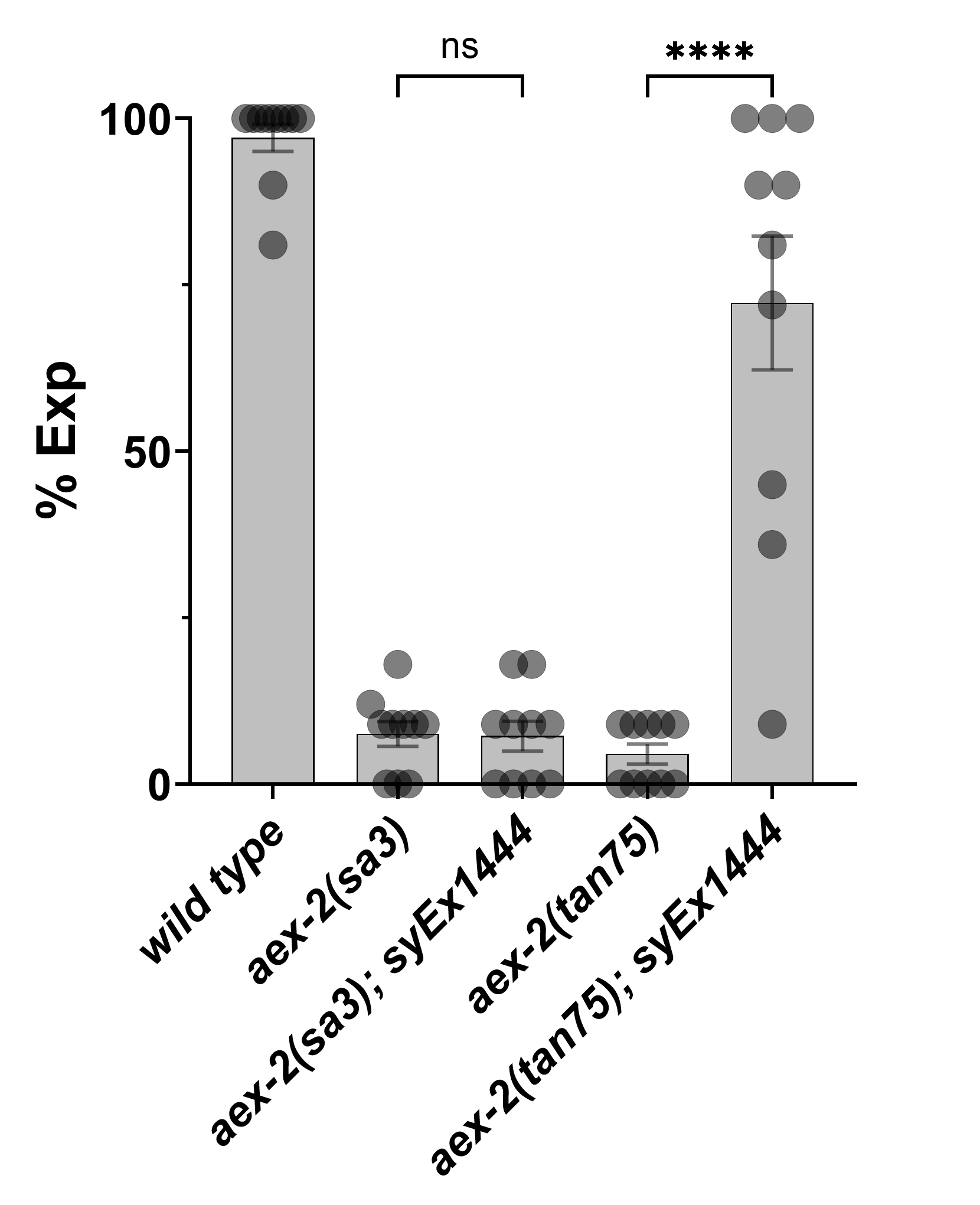

Supplement: S3 Fig — The expulsion defects in aex-2(tan75[cGAL GT]) mutants can also be rescued with a multi-copy 15xUAS::aex-2(+) extrachromosomal array effector (syEx1444). This rescue is not observed in aex-2(sa3); syEx1444 animals. Each dot represents a single worm (N = 10 worms). Mean ± SEM is shown. ns, not significant; ****, p<0.0001. One-way ANOVA with post-hoc Tukey HSD. (TIF) [file pgen.1011541.s003.tif]

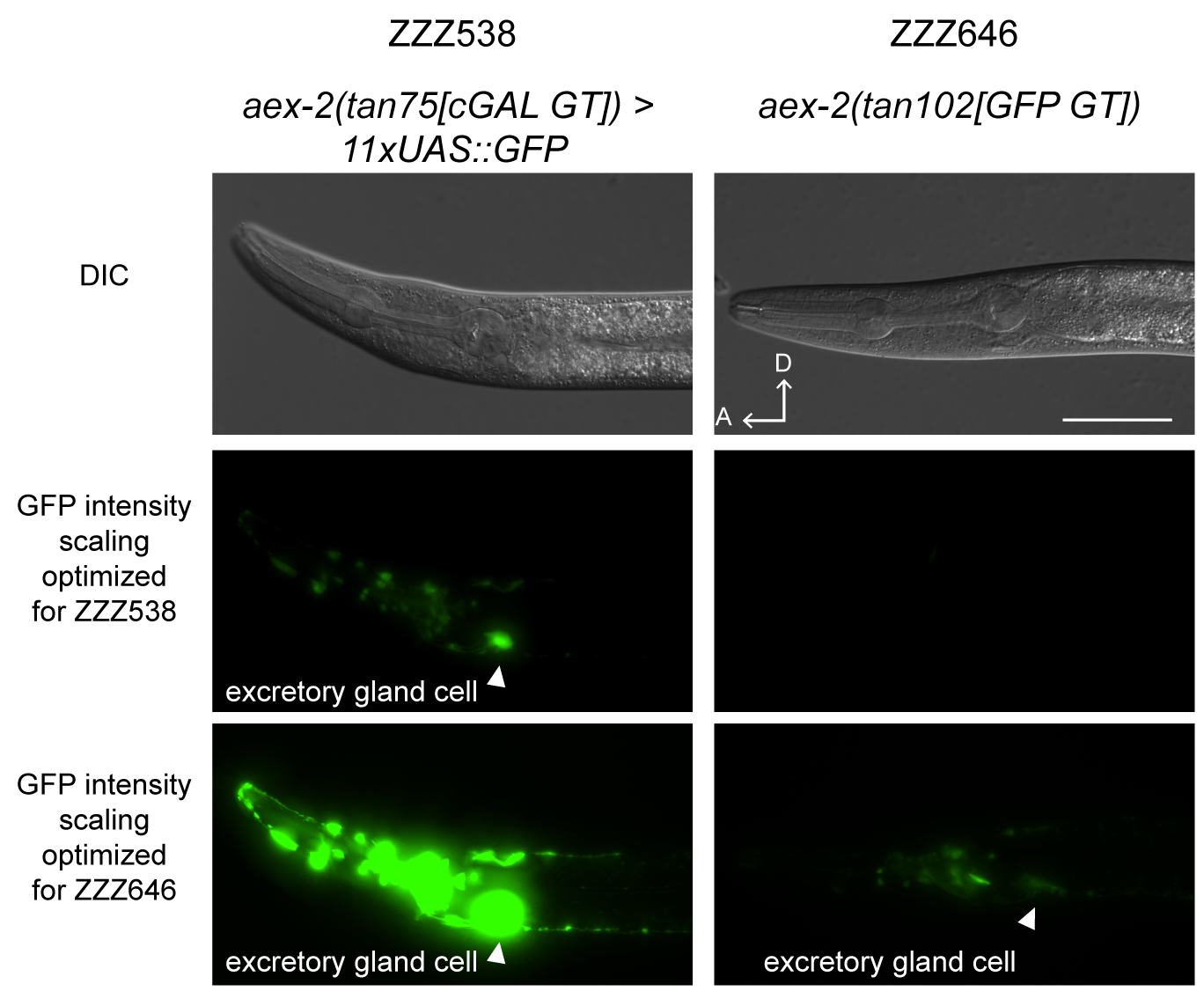

Supplement: S4 Fig — ZZZ538 is tanSi34[11xUAS::GFP]; aex-2(tan75[cGAL GT]) double homozygote, and ZZZ646 is aex-2(tan102[GFP GT]). Both cGAL in the tan75 allele and GFP in the tan102 allele were single-copy transgenes inserted at the same location in the third intron of aex-2. DIC images of the head region of both strains are shown (top panel). When digitally optimizing the look-up table to observe GFP expression in ZZZ538, no GFP expression is observed in ZZZ646 animals (middle panel). Alternatively, when optimizing the look-up table to observe dim GFP expression in ZZZ646, the GFP expression in ZZZ538 becomes overexposed (bottom panel). Arrowheads indicate the excretory gland cell. Scale bar is 50 μm. A, anterior; D, dorsal. (TIF) [file pgen.1011541.s004.tif]

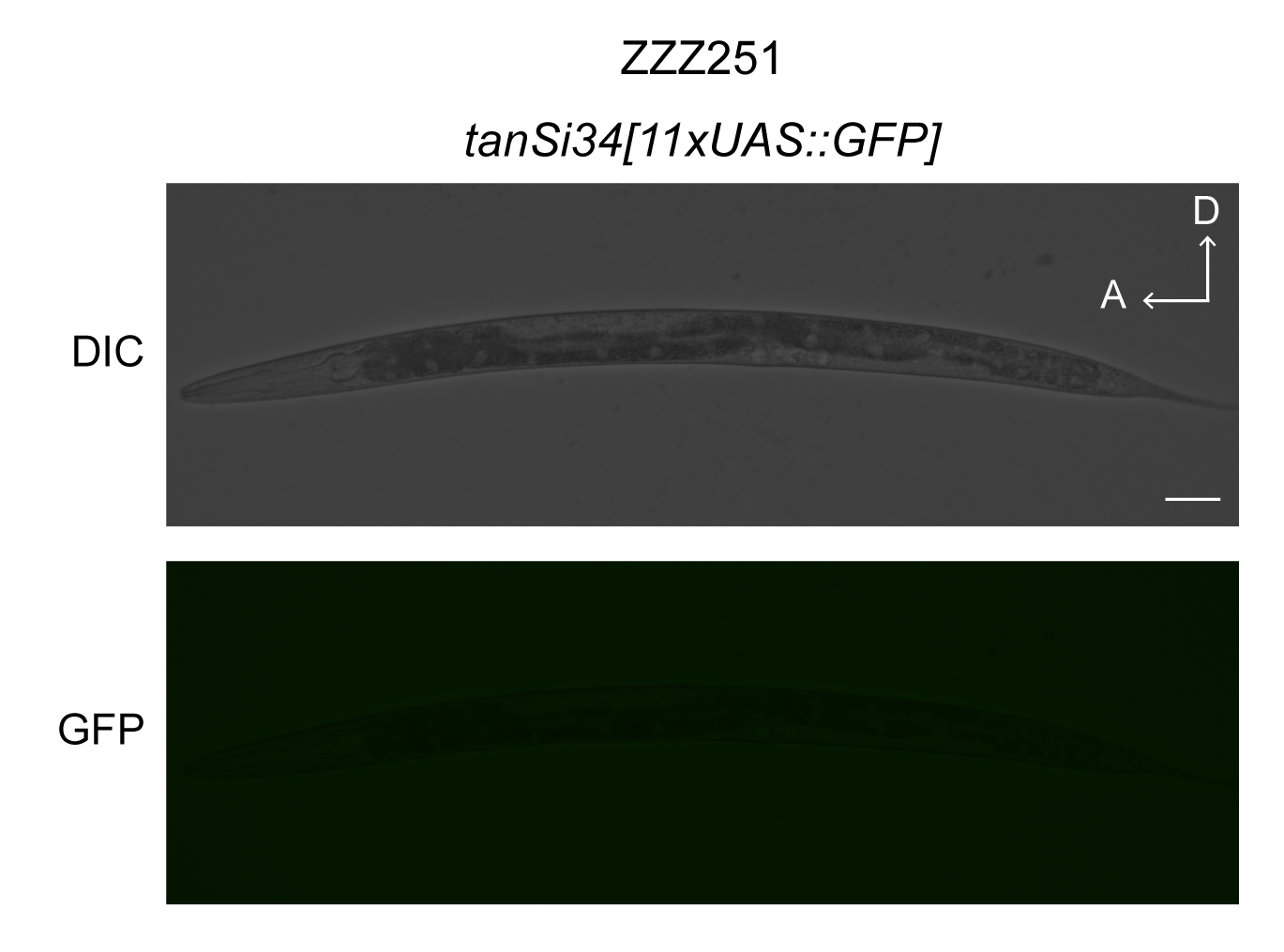

Supplement: S5 Fig — DIC and fluorescent images of the single-copy GFP effector strain ZZZ251 (tanSi34[11xUAS::GFP]). Scale bar is 50 μm. A, anterior; D, dorsal. (TIF) [file pgen.1011541.s005.tif]

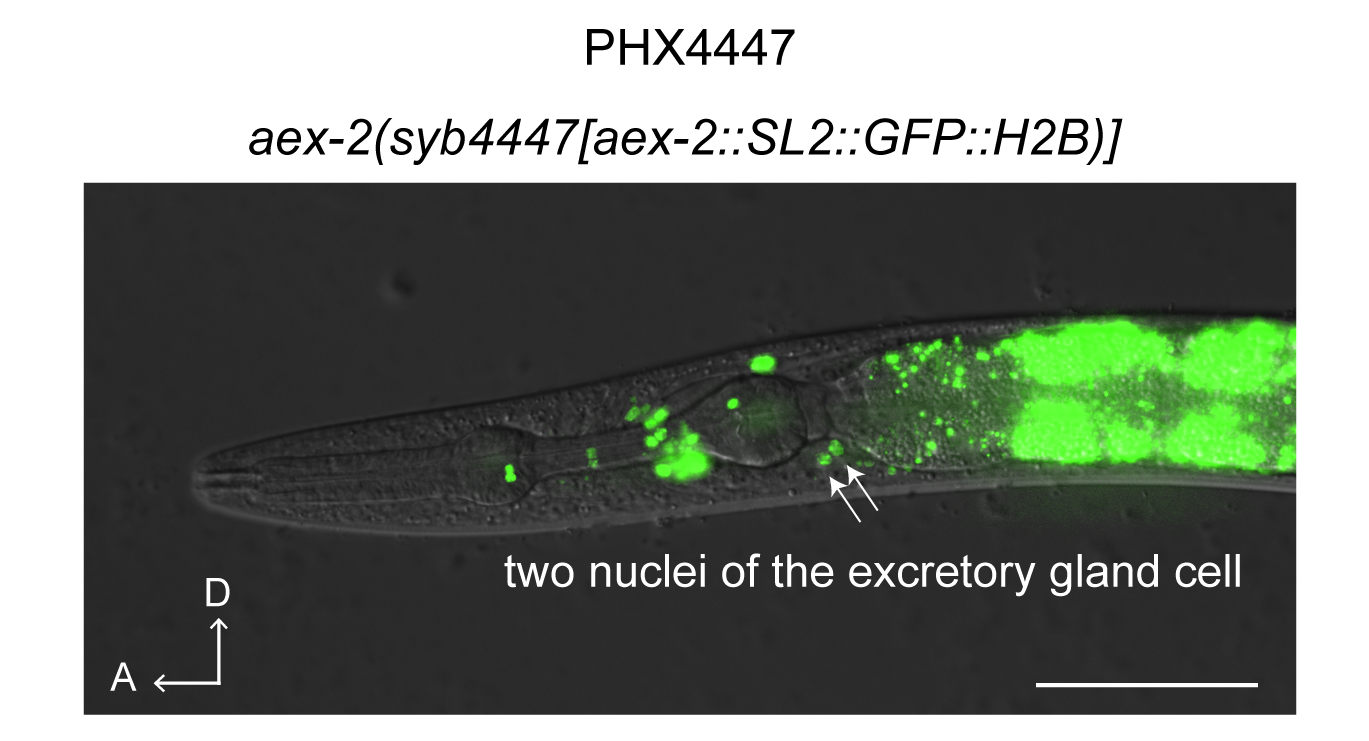

Supplement: S6 Fig — Merged image of DIC and fluorescent images of the strain PHX4447, a transcriptional reporter strain of aex-2, in which a trans-spliced sequence encoding a nuclear localized GFP fusion (SL2::GFP::H2B) is inserted right after the endogenous aex-2 locus. Very weak GFP fluorescence is observed in the two nuclei of the binucleate excretory gland cell, as indicated by two white arrows. Scale bar is 50 μm. A, anterior; D, dorsal. (TIF) [file pgen.1011541.s006.tif]
