# Supplementary material for: A versatile site-directed gene trap strategy to manipulate gene activity and control gene expression in Caenorhabditis elegans
Source: PLoS Genet. 2025 Jan 22;21(1):e1011541. doi: 10.1371/journal.pgen.1011541 (PMC11753634; doi:10.1371/journal.pgen.1011541)
Supplement: S7 Fig — The most effective cross scheme is scheme 1, in which the males are double homozygous for the cGAL gene trap (cGAL GT) and the germline-expressing Flp transgene (bqSi711). Hygromycin serves as a positive selection marker for both the successful RMCE single-copy insertion as well as the array with the RMCE donor constructs; histamine serves as a negative selection marker for the array. Refer to see Materials and Methods for more details. (PDF) [file pgen.1011541.s007.pdf]

Scheme 1

1. Generate cGAL GT(*tan#*); *bqSi711* IV ⚥
2. Heat shock to induce cGAL GT(*tan#*); *bqSi711* ♂
3. Set up cross

RMCE ex-array (*tanEx#*) ⚥ x cGAL GT(*tan#*); *bqSi711* IV ♂

↓ Place at RT and wait for next generation (~3 days).

cGAL GT(*tan#*)/+; *bqSi711*/+; RMCE ex-array ⚥

↓ Add hygromycin to enrich for ex-arrays and wait for next generation (~3 days).

↓ Add histamine to enrich for RMCE progeny and select against ex-array.
Wait for next generation (~3-5 days).

RMCE progeny (*tan# tan#*)/+; *bqSi711*/+ ⚥

Scheme 2

1. Generate cGAL GT(*tan#*); *bqSi711* IV ⚥
2. Set up cross

cGAL GT(*tan#*); *bqSi711* ⚥ x N2 ♂

↓ Pick green ♂ (cross progeny with germline mNeonGreen)

RMCE ex-array (*tanEx#*) ⚥ x cGAL GT(*tan#*)/+; *bqSi711*/+ ♂

↓ Place at RT and wait for next generation (~3 days).

cGAL GT(*tan#*)/+; *bqSi711*/+; RMCE ex-array ⚥

↓ Add hygromycin to enrich for ex-arrays and wait for next generation (~3 days).

↓ Add histamine to enrich for RMCE progeny and select against ex-array.
Wait for next generation (~3-5 days).

RMCE progeny (*tan# tan#*)/+; *bqSi711*/+ ⚥

Scheme 3

1. Heat shock to induce *bqSi711* ♂
2. Set up cross

cGAL GT(*tan#*) ⚥ x *bqSi711* ♂

↓ Pick green ♂ (cross progeny with germline mNeonGreen)

RMCE ex-array (*tanEx#*) ⚥ x cGAL GT(*tan#*)/+; *bqSi711*/+ ♂

↓ Place at RT and wait for next generation (~3 days).

cGAL GT(*tan#*)/+; *bqSi711*/+; RMCE ex-array ⚥

↓ Add hygromycin to enrich for ex-arrays and wait for next generation (~3 days).

↓ Add histamine to enrich for RMCE progeny and select against ex-array.
Wait for next generation (~3-5 days).

RMCE progeny (*tan# tan#*)/+; *bqSi711*/+ ⚥

For all three schemes, single out to find non-green homozygous Rol for the next generation.
Then, HS to remove SEC to get final RMCE knock-in lines.
