# Supplementary material for: A versatile site-directed gene trap strategy to manipulate gene activity and control gene expression in Caenorhabditis elegans
Source: PLoS Genet. 2025 Jan 22;21(1):e1011541. doi: 10.1371/journal.pgen.1011541 (PMC11753634; doi:10.1371/journal.pgen.1011541)
Supplement: S1 Table — (PDF) [file pgen.1011541.s008.pdf]

Table S1. Efficiency of gene trap swapping through RMCE by genetic crossing

**Cross Scheme***	**Loci**	**cGAL GT strain (males)**	**RMCE ex-array donor strain**	**Temp**	**Number of cross plates**	**Number of cross plates with RMCE progeny present****	**Efficiency (%) *****
Scheme 1	*aex-2*	ZZZ322	ZZZ630 (Tet-Off)	25°C	10	8	80
	aex-2	ZZZ322	ZZZ700 (QF)	RT	5	5	100
	aex-2	ZZZ322	ZZZ701 (QF2)	RT	5	2	40
	aex-2	ZZZ322	ZZZ703 (LexA)	RT	5	2	40
Scheme 2	*unc-47*	ZZZ635/+	ZZZ700 (QF)	RT	5	2	40
	unc-47	ZZZ635/+	ZZZ701 (QF2)	RT	5	3	60
	unc-47	ZZZ635/+	ZZZ703 (LexA)	RT	10	1	10
	unc-47	ZZZ635/+	ZZZ630 (Tet-Off)	RT	6	3	50
	unc-47	ZZZ635/+	ZZZ840 (LexA)	RT	5	3	60
	unc-47	ZZZ635/+	ZZZ1092 (Tet-On)	RT	5	1	20
	unc-47	ZZZ635/+	ZZZ1093 (Tet-On)	RT	5	2	40
Scheme 3	*inx-16*	ZZZ532/+; BN711/+	ZZZ738 (cGAL)	RT	5	3	60
	inx-16	ZZZ533/+; BN711/+	ZZZ738 (cGAL)	RT	5	1	20

*, see S7 Fig for the details of the cross schemes.

**, RMCE progeny refer to healthy roller animals that are resistant to both hygromycin and histamine in the cross plates.

***, efficiency (%) is the ratio of the number of cross plates with RMCE progeny present over the number of cross plates.
