# Supplementary material for: A versatile site-directed gene trap strategy to manipulate gene activity and control gene expression in Caenorhabditis elegans
Source: PLoS Genet. 2025 Jan 22;21(1):e1011541. doi: 10.1371/journal.pgen.1011541 (PMC11753634; doi:10.1371/journal.pgen.1011541)
Supplement: Attachment — Submitted filename: Reviewer 1_comments.docx [file pgen.1011541.s013.docx]

The manuscript by Khan et al. developed a GAL4-UAS system-based gene trap system in *C. elegans*. The strategy will allow for generating mutations that simultaneously provide genetic access to the cells expressing the altered gene. With their design, the cGAL gene trap is inserted into an intron of a gene, leading to the truncation of the target protein and the expression of cGAL in the cells that mimics the regular expression pattern of the interrupted gene. By combining with different UAS effector lines, one can analyze the gene expression pattern, rescue the gene-trap mutant by re-expressing the protein in the cells where it is endogenously expressed, or manipulate the cells that express the target gene to reveal its function. The authors used the strategy in the manuscript to successfully generate gene-trap *C. elegant lines* for *aex-2*, *unc-47*, and *inx-16*. The FRT site and the reverse FRT3 site flank the gene trap enabling the conversion of the gene trap to other bipartite expression systems such as QF, QF2, LexA, Tet-Off, and Tet-on systems via FLP recombinase-mediated RMCE. The authors also found that the efficiency of RMCE by genetic crossing is comparable to or even higher than RMCE via microinjection. The find alone is exciting and beneficial for the *C. elegan*s community to explore more potential applications. Overall, the gene trap described in the manuscript is a powerful and versatile tool in *C. elegans* research.

The manuscript is well-written, and the data is convincing. However, the manuscript should address more of the need for such a tool for the *C. elegans* community, the potential applications for the gene trap tool, and its advantages over the traditional methods. The manuscript should be improved by answering the following comments and questions to strengthen the proposed claims.

**Major comments:**

1. CRISPR-Cas9 gene editing is more efficient for deletions or small insertions over larger insertions (PMID: 37254814). What is the efficiency of generating the gene trap lines? CRISPR efficiency may be different from case by case. Could the authors comment on the general efficiency of generating the gene trap lines in this manuscript? CRISPR efficiency could be a limitation factor in choosing the gene trap over traditional approaches such as gene deletion mutants and tissue-specific promoter-cDNA overexpression systems.
2. Follow up with the previous question. The authors showed the possibility of RMCE by genetic cross, which is very exciting. Have the authors considered or tested a two-step gene trap strategy? Can the gene trap lines be generated by generating a CRISPR knock-in strain with the FRT/FRT3 sites flanking the SA+short exon + 3Xstop (small insertion, easy to CRISPR)? Then, it is crossed with a strain with a gene trap flanked by FRT/FRT3 to switch it into a gene trap strain. In this way, one will generate a mutant expressing a trucked protein with high efficiency and have the option to use the gene trap system by genetic crosses.
3. Overall, the gene trap's advantage needs to be addressed more clearly. In other systems, such as Arabidopsis, the gene trap has been successfully used for forward genetic screen to visualize the mutant phenotype and the gene expression pattern simultaneously. Could the authors discuss the potential and limitations of such an application of the gene trap in *C. elegans*?
4. In Figure 2B, is the *aex-2(tan75)/+* statistically insignificant from wild-type animals? If not, is it expected, and why? Same for the *syIs371/+ aex-2(tan75)/+* strain in Figure 2D.
5. Is the excretory gland cell expression of *aex-2* also true in the gfp effector strain ZZZ538 in Figure S4? If so, it may be worth mentioning it in the text. To clarify if the gene trap system can reveal unknown expression patterns (probably through amplifying the signal) missed by the traditional methods or if the gene trap could lead to ectopic expression of the reporter. It could be tested by endogenously tagging AEX-2 with GFP (without the-2 UTR or the gene trap) to see if AEX-2::GFP is present in the excretory gland cell.
6. Line 222-223. By reading the sentence, it appears that the goal is to study the target gene's function in the neurons expressing it. To do it, one would expect to compare between *aex-2(tan75)*/*aex-2(tan75)* and *aex-2(tan75)/+* in a particular process. However, by overexpressing HisCl, the neurons expressing *aex-2* are silenced. The results support the idea that crossing *aex-2(tan75)* with certain effector lines can manipulate the neurons/cells expressing *aex-2*. However, it does not reveal the exact function of *aex-2*. I am wondering if crossing aex-2(tan75) with a calcium reporter UAS::GCaMP6s::SL2::mKate2, and compare the calcium between *aex-2(tan75)/aex-2(tan75)* and aex-2(tan75)/+ (PMID: 27992408) would be more suitable for this purpose. This proposal assumes that heterozygous and homozygous *aex-2(tan75)* drive similar expression levels of the effector reporter, which may not true. If not, a better control must be designed to study gene function using the gene trap systems.
7. In Figure 4C, the LexA GT strain seems to have less fluorescent signal. Is it true? Could the authors comment on it?

**Minor comments:**

8. In Figures 1, 2, and 4, the yellow color and text of FRT are very hard to see on a white background. It might be better to change it to a different color.
9. The existing effector strains could be an advantage of using the gene trap system. Maybe it is worth listing the existing effector strains (such as <https://wormlab.caltech.edu/projects/cgal>) to emphasize the power of the tool kit.
10. In the introduction, lines 76-77, the authors wrote, "the resultant alleles cannot be directly used to control the expression of other genes." This statement sounds like it came from nowhere and is not logically smoothly connected with the first half of the sentence. Why does it need to be used to control the expression of other genes? I suggest reorganizing the sentences.
11. In the introduction, lines 96-97, the authors mentioned that no genetic approach exists for *C. elegans* researchers to disrupt gene activity and simultaneously provide genetic access to the cells. It would be more appealing to address the significance of such an approach (or the limitations of the current tools).
12. The authors mentioned different bipartite expression systems available for *C. elegans* research. Would it be worthwhile to include a brief overview of the systems and the limitations and advantages of each one to enhance the advantage of the versatility of the gene trap?
