# Supplementary material for: A versatile site-directed gene trap strategy to manipulate gene activity and control gene expression in Caenorhabditis elegans
Source: PLoS Genet. 2025 Jan 22;21(1):e1011541. doi: 10.1371/journal.pgen.1011541 (PMC11753634; doi:10.1371/journal.pgen.1011541)
Supplement: Attachment — Submitted filename: cGAL gene trap response to reviewers to resubmit.docx [file pgen.1011541.s014.docx]

Reply to reviewers (PGENETICS-D-24-01003)

We thank the reviewers for their insightful and constructive comments and appreciate their overall enthusiasm for our manuscript. We provide detailed responses to the reviewers’ comments and have made the corresponding changes to the revised manuscript (*detailed below in blue*). We believe the revision has improved the overall quality of the manuscript.

Reviewer#1:

Major comments:

1. CRISPR-Cas9 gene editing is more efficient for deletions or small insertions over larger insertions (PMID: 37254814). What is the efficiency of generating the gene trap lines? CRISPR efficiency may be different from case by case. Could the authors comment on the general efficiency of generating the gene trap lines in this manuscript? CRISPR efficiency could be a limitation factor in choosing the gene trap over traditional approaches such as gene deletion mutants and tissue-specific promoter-cDNA overexpression systems.

We are sorry that we only mentioned relevant information in the supplemental information but not in the main manuscript. As the cGAL gene trap cassette is relatively large, we used the well-established CRISPR knock-in technique with SEC selection for inserting large DNA fragments in defined loci in C. elegans genome, with insertion efficiency ranging from 2.5%-12.5% (Dickinson, Genetics, 2015). The use of the SEC transgenic marker requires minimal labor to screen for positive gene trap knock-in animals. In addition, compared to gene deletion mutants using traditional approaches, the cGAL gene trap lines are advantageous as they not only can serve as loss-of function alleles but also can be combined with many UAS effectors by simple genetic crossing for various experimental perturbations as shown in the manuscript. To specifically address the KI efficiency comment, we move the supplemental information into the Materials and Methods of the revised manuscript.

Page 22, “Next, the solution was injected into ~40 young adult N2 (Bristol strain) worms (P0) per cGAL gene trap knock-in, with 2 P0 per plate, and the injected worms were placed at 25°C”

2. Follow up with the previous question. The authors showed the possibility of RMCE by genetic cross, which is very exciting. Have the authors considered or tested a two-step gene trap strategy? Can the gene trap lines be generated by generating a CRISPR knock-in strain with the FRT/FRT3 sites flanking the SA+short exon + 3Xstop (small insertion, easy to CRISPR)? Then, it is crossed with a strain with a gene trap flanked by FRT/FRT3 to switch it into a gene trap strain. In this way, one will generate a mutant expressing a trucked protein with high efficiency and have the option to use the gene trap system by genetic crosses.

We thank this reviewer for this thoughtful comment. It should be potentially feasible to use the suggested two-step protocol, but we haven’t directly tried it before for a couple of reasons. First, it will take more time (2-3x) using the two-step strategy, as it requires a CRIPSR experiment and multiple crosses to bring the germline expressing transgene and the extrachromosomal array with gene trap flanked by FRT/FRT3 into the CRISPR allele. Second, as the 34-bp long FRT and FRT3 are almost identical except 4 bp within the 8-bp core sequence in the center, a single-strand repair oligo with FRT and reverse FRT3 sequences may create a hairpin-like DNA structure that may impede its ability to serve as an effective repair template for CRISPR knock-in experiments. Nonetheless, in certain conditions, e.g., the targeted genomic locus is very challenging to insert the large cGAL gene trap cassette using CRIPSR knock-in with SEC, the suggested two-step strategy can potentially be a good option to try.

3. Overall, the gene trap's advantage needs to be addressed more clearly. In other systems, such as Arabidopsis, the gene trap has been successfully used for forward genetic screen to visualize the mutant phenotype and the gene expression pattern simultaneously. Could the authors discuss the potential and limitations of such an application of the gene trap in *C. elegans*?

We apologize for not explaining this clearly in the original manuscript. We have added subtitles in the Discussion section in the revised manuscript to clearly show the advantages, limitations, and potential of the cGAL gene trap system. (See Page 17-19)

4. In Figure 2B, is the *aex-2(tan75)/+* statistically insignificant from wild-type animals? If not, is it expected, and why? Same for the *syIs371/+ aex-2(tan75)/+* strain in Figure 2D.

It is statistically insignificant. This is expected, as *aex-2(tan75)* is a recessive loss-of-function allele; thus, one wild-type copy of *aex-2* would be sufficient for its function.

5. Is the excretory gland cell expression of *aex-2* also true in the gfp effector strain ZZZ538 in Figure S4? If so, it may be worth mentioning it in the text. To clarify if the gene trap system can reveal unknown expression patterns (probably through amplifying the signal) missed by the traditional methods or if the gene trap could lead to ectopic expression of the reporter. It could be tested by endogenously tagging AEX-2 with GFP (without the-2 UTR or the gene trap) to see if AEX-2::GFP is present in the excretory gland cell.

We thank this reviewer for this thoughtful comment. We easily saw bright excretory gland cell expression of *aex-2* cGAL gene trap in the strain ZZZ538 and could barely see its expression in the aex-2 GFP genet trap line (ZZZ646) in Fig S4; the different intensity is likely due to the amplification of the cGAL-UAS system. As suggested, we also examined an endogenous aex-2 reporter strain available from CGC (PHX4447 aex-2(syb4447 [aex-2::SL2::GFP::H2B)] X), and observed very dim GFP signal in the nucleus of the excretory gland cell, further confirming that the cGAL gene trap system can reveal endogenous expression pattern that would be otherwise missed due to dim or no expression using traditional transcriptional reporters. Accordingly, we have labelled the excretory gland cell in Fig S4 and added the new data of PHX4447 in Fig S6.

Fig S6. *aex-2* is expressed in the excretory gland cell.

6. Line 222-223. By reading the sentence, it appears that the goal is to study the target gene's function in the neurons expressing it. To do it, one would expect to compare between *aex-2(tan75)*/*aex-2(tan75)* and *aex-2(tan75)/+* in a particular process. However, by overexpressing HisCl, the neurons expressing *aex-2* are silenced. The results support the idea that crossing *aex-2(tan75)* with certain effector lines can manipulate the neurons/cells expressing *aex-2*. However, it does not reveal the exact function of *aex-2*. I am wondering if crossing aex-2(tan75) with a calcium reporter UAS::GCaMP6s::SL2::mKate2, and compare the calcium between *aex-2(tan75)/aex-2(tan75)* and aex-2(tan75)/+ (PMID: 27992408) would be more suitable for this purpose. This proposal assumes that heterozygous and homozygous *aex-2(tan75)* drive similar expression levels of the effector reporter, which may not true. If not, a better control must be designed to study gene function using the gene trap systems.

We apologize for not explaining the logic of the experiment clearly in the original manuscript. It has been previously shown that activation of AEX-2 by its ligand NLP-40 activates the AEX-2-expressing neurons (in particular AVL and DVB neurons) to trigger the expulsion step (Wang et at., Current Biology, 2013). Our intention was to show that cGAL gene trap alleles offer a convenient way to manipulate the target-gene-expressing neurons to infer the function of these neurons. We used the *aex-2* cGAL gene trap and a UAS::HisCl effector as an example to show the feasibility of such an application. To address the confusion, we have changed the corresponding texts in the revised manuscript.

Page 11: “Next, we tested if cGAL gene trap lines can be used to manipulate the neurons that express the target gene to reveal the function of these neurons. Activation of the two GABAergic neurons, AVL and DVB, by the AEX-2 signaling triggers the expulsion step [22,23]. Thus, we determined if we could use the aex-2 cGAL gene trap line to inhibit aex-2-expressing neurons and tested if silencing these neurons phenocopies aex-2 mutants. To this end, we crossed the cGAL gene trap line aex-2(tan75) with the transgene syIs317, a previously validated 15xUAS::HisCl1::SL2::gfp effector line [14], and quantified expulsion defects (Fig 2D).”

7. In Figure 4C, the LexA GT strain seems to have less fluorescent signal. Is it true? Could the authors comment on it?

Thanks for pointing this out. We re-examined our images and observed LexA GT strain also reliably expressed GFP in the head GABAergic neuron. For unknown reasons, in LexA GT strain, the GFP signal in GABAergic neurons in the head region is much weaker than the signal in those neurons along the ventral cord, which results in the impression that the LexA GT strain seems to have less neurons with fluorescent signal. While the bipartite expression systems described in the manuscript all works as single-copy transgene as gene trap, the relative performance of each of these bipartite expression systems can be potentially influenced by the inserted genome locus. We have modified the texts in revised manuscript.

Page 15: “When combined with their corresponding GFP effector lines, these new gene traps also drive GFP expression in the GABAergic neurons (Fig 4C), largely similar to the unc-47 cGAL gene trap (Fig 3E), suggesting these gene trap lines all reflect the endogenous expression pattern of unc-47. For reasons unknown, GFP signal in the GABAergic neurons in the head region of the unc-47 LexA gene trap appeared to be much dimmer than the signal in those neurons along the ventral cord.”

Minor comments:

1. In Figures 1, 2, and 4, the yellow color and text of FRT are very hard to see on a white background. It might be better to change it to a different color.

As suggested, we have changed the outline and color of FRT to enhance the contrast.

2. The existing effector strains could be an advantage of using the gene trap system. Maybe it is worth listing the existing effector strains (such as <https://wormlab.caltech.edu/projects/cgal>) to emphasize the power of the tool kit.

We apologize for not explaining this clearly in the original manuscript. We have modified the texts to include specific references for existing effector strains and highlight the number of available effector strains and their potential application for various experimental purposes.

Page 19: “The cGAL gene trap lines can easily be combined with growing numbers of available UAS effector lines, which includes more than 30 validated UAS effector lines for manipulating neuron activity, ablating cells, expressing various fluorescent reporters, and perturbing specific signaling transduction pathways [14,37,38].”

3. In the introduction, lines 76-77, the authors wrote, "the resultant alleles cannot be directly used to control the expression of other genes." This statement sounds like it came from nowhere and is not logically smoothly connected with the first half of the sentence. Why does it need to be used to control the expression of other genes? I suggest reorganizing the sentences.

We are sorry for the confusion. We intended to convey the limitations of the current genetic tools to generate mutant alleles—existing tools can create mutant alleles, but these alleles cannot be directly combined with bipartite expressions systems to control transgene expression. We have edited the texts to make it clearer.

Page 4-5: “While these powerful tools can manipulate endogenous gene activity by creating mutations for functional genetic studies, the resultant alleles cannot be directly used for transgene control.”

4. In the introduction, lines 96-97, the authors mentioned that no genetic approach exists for *C. elegans* researchers to disrupt gene activity and simultaneously provide genetic access to the cells. It would be more appealing to address the significance of such an approach (or the limitations of the current tools).

We apologize for not explaining this well in the original manuscript. Such an approach will dramatically increase the efficiency for genetic studies to understand what the targeted gene does and where it functions. We have added the following texts to the revised manuscript.

Page 5-6: “However, there is no genetic approach for C. elegans researchers to disrupt gene activity and simultaneously provide genetic access to cells that express the disrupted gene for controlling transgene expression; such a genetic approach will dramatically increase the efficiency of genetic studies to reveal the function and site of action of the gene of interest.”

5. The authors mentioned different bipartite expression systems available for *C. elegans* research. Would it be worthwhile to include a brief overview of the systems and the limitations and advantages of each one to enhance the advantage of the versatility of the gene trap?

We appreciate this comment. While we agree that a comparison of advantages and limitations among each of bipartite expression systems can be very useful, such an overview would fit better in manuscripts that aim to improve the functionalities of different bipartite expression systems. Rather, our study addresses the versatility of our cGAL gene trap strategy on its convenience for various experimental perturbation when combined with different effectors and its extensibility by easy swapping to other bipartite expression systems.

Reviewer #2:

One general comment about the gene trap design that the authors do not mention. As described, the splice acceptor and three-frame stop codons should truncate the protein. However, the downstream gpd-2/3 operon would be expected to do the same. The operon sequence leads to transcriptional termination and re-initiation. I do not know of any examples of operon sequences being "ignored," for example, for alternative splicing, so in effect, the authors have a "double termination" system. This may be worth including in the text, especially as the authors discuss the insertion site of the cassette relative to an endogenous splice site. It should, in principle, not matter given their design - including insertions into exons.

We thank this reviewer for this thoughtful comment. We agree with this reviewer that when even if cGAL gene trap is inserted into exons, the cGAL gene trap should still work, as the three-frame stop codons should also create a truncated protein, no matter the endogenous splice site or the added splice site is used for splicing to produce the mRNA. Indeed, our preliminary result from a different project showed that inserting the cGAL gene trap in the exon can also work as predicted (unpublished results, data not shown).

We have added a paragraph in the Discussion to discuss the rationale for the choice of the insertion site of the cGAL gene trap.

Pages 16-17: “Considerations for the insertion site of the cGAL gene trap cassette

In our study, we tested our cGAL gene trap design by inserting the cGAL gene trap cassette in introns of the target genes and our results showed that gene trap works robustly for all the genes tested. For example, both inx-16(tan99) and inx-16(tan174), with the cGAL gene trap cassette inserted at different introns, showed the same mutant phenotype and expression pattern (Fig 3). We recommend considering the following factors when selecting an insertion site to generate a new gene trap for the gene of interest: 1) select an intron of your target gene with a specific guide RNA sequence (http://genome.sfu.ca/crispr/search.html, [30]) to insert the gene trap cassette into the genome via CRISPR; 2) if the main goal is to use the gene trap as a “promoter trap” by only focusing on the enhancer and regulatory elements in the promoter, then choose an insertion site within an intron closer to the start of the gene; 3) if the main goal is to use the gene trap to capture as many regulatory elements within the introns of the gene in addition to the promoter, then choose an insertion site within an intron closer to the end of the gene; 4) if the main goal is to use gene trap as a loss-of-function mutant, then choose an insertion site upstream of the coding sequence for critical domains of the target gene. Furthermore, as the cGAL gene trap cassette contains an added splice acceptor followed by multiple stop codons in all three reading frames, in principle, the gene trap strategy should also work even if the cassette is inserted into exons of target genes.”

Suggested changes and Specific comments:

1. I would mainly ask the authors to clarify and make the following additions to the text:
The methods mention validation of the insertion sites by PCR (and even a short discussion of using Oxford Nanopore to validate). There is no mention of the validation in the text. Please include a brief sentence in the results about whether all insertions were validated. Did the authors ever observe off-target insertions?

As suggested, we have added the following sentence to the revised manuscript about the validation of the insertion sites. We don’t really see off-target insertions.

Page 15: “Successful swaps to transcriptional factors from other bipartite expression systems in these new gene trap lines were validated using Oxford Nanopore linear amplicon sequencing of PCR products containing the section between the FRT site and the reverse FRT3 site as well as a few hundred base pairs of upstream and downstream flanking genomic DNA sequences.”

2. Lines 206 - 213: Could the authors comment more exhaustively on the expression in the gland cells? There is a slight discrepancy with previously characterized expression patterns (although expression is consistent with more recent single-cell data but then the expression is higher than expected). Do the authors think this has to do with amplification of the fluorescence signal using the 11x UAS::mScarlet construct? For example, the minimal promoter used in the 11x UAS::mScarlet construct? Or could this be caused the 3' UTR regulation, either of the tbb-2 3' UTR in the cGal GT construct or the 11x UAS::mScarlet construct? Understanding how accurately the expression patterns reflect the endogenous expression is important for the utility of using the gene-trap cassette for further experiments.

We thank this reviewer for this thoughtful comment. We believe that the cGAL gene trap can better reflect endogenous gene expression pattern compared to classic promoter-GFP transcriptional fusion constructs, as gene trap is under the control of the endogenous promoter and other regulatory elements in upstream introns. The amplification by the cGAL/UAS system not only makes it easier to see expression previously reported but also helps reveal additional cells expressing low levels of the target gene that would otherwise to too dim to be observed using a traditional transcriptional/translational reporter. We don’t believe that the observation of aex-2 expression in the excretory gland cell is due to the tbb-2 3’UTR or the minimal promoter (pes-10) in the effector line, because we did not see expression in the excretory gland cell in the inx-16 and unc-47 cGAL gene trap lines, when they were combined to the same UAS GFP effectors. We also confirmed the expression of aex-2 in the excretory gland cell using an endogenous aex-2 reporter strain available from CGC (PHX4447 aex-2(syb4447 [aex-2::SL2::GFP::H2B)] X). See Fig S6 above and in the revised manuscript.

1. Lines 217 - 218: The signal amplification from the 11x UAS::GFP construct is an important consideration. Could the authors comment and/or characterize the following:

A. Approximately how much amplification do they observe? They only mention different look-up tables, but it would be useful to have a "solid number" for how much signal amplification is observed, which should be easily inferred from the images and the microscopy settings on the already included images.

We estimated about 8-fold amplification, but the amplification for gene trap can potentially be locus-specific or cell-specific. For the aex-2 cGAL gene trap lines, we can easily see the bright GFP expression under a standard dissecting fluorescence microscope (Leica M165 FC), when combined with tanSi34, a single-copy UAS::GFP effector line but can barely see GFP signal the aex-2 GFP gene trap line.

B. A second question concerns how many cells they see expression in. From the images in Fig. S4, it is difficult for the reader to see if the patterns are "similar" but just have different intensities. This is, of course, a difficult question to answer, but it would be useful for the reader to know how many cells are expected to express a-2 based on single-cell data and whether the patterns are consistent with this.

Due to the expression of aex-2 in many neurons packed in the head region and the various intensities between different cells, we have not been able to exactly count how many cells we see expression in. We observed expression in neurons (AVL and DVB) where aex-2 functions to regulate the expulsion step and cells with distinct locations (such as HMC and the excretory gland cells). Based on other gene trap lines (*inx-16* (intestine) and *unc-47* (GABAergic neurons), the expression patterns from the cGAL gene traps often are consistent with previous reports.

3. Lines 329 - 331: I agree that the gene trap cassette is versatile. However, could the authors comment on if and how the gene trap would be used in genes with lethal or sterile phenotypes? Are there ways to maintain the gene trap insertion for alleles that need to be heterozygous? The authors use the self-excising cassette (SEC), which contains a positive selection marker (HygroR). Using the SEC is convenient but would appear to be limiting for genes with a severe loss-of-function phenotype because the positive selection is removed before the genetically modified animals are "used" in experiments. Unless I am missing an important point, I would suggest including this in the list of limitations that comes immediately after in the discussion.

We thank this reviewer for this thoughtful comment. We are sorry that we did not mention this specific scenario in the original manuscript. The cGAL gene trap should still be applied to lethal or sterile genes. Like the genes we reported in the manuscript, it should be straightforward to generate viable heterozygous Rol + HygR worms (from SEC) using our cGAL gene trap strategy for sterile or lethal genes. These animals then can be directly crossed to a UAS::GFP effector line (such as *tanSi34*). Eventually, the worms with Gene trap/+; UAS::GFP/UAS::GFP can be used to observe gene expression and at the same time serve as a balancer for the lethal/sterile gene trap allele (even after the SEC is removed). This is likely feasible, as in our hands, for all the cGAL gene trap lines generated in our lab so far, when combined with the single-copy UAS GFP effector (*tanSi34*), we can observe bright GFP signal using a standard fluorescence dissecting microscope.

4. Lines 351 - 357: I am not sure I understand the logic of the discussion of the location of the enhancers relative to where the gene trap is inserted. If an enhancer is located in an intron downstream of the gene trap insertion (or further away), the enhancer could presumably still function to regulate transcription at the TSS. It is true that any perturbation of the genomic locus (including a "normal" N or C terminal gfp tag) can potentially disrupt transcriptional regulation, but that is difficult to predict based on the location. Perhaps the authors could elaborate on what exactly they mean.

We are sorry for the confusion. As our cGAL gene trap cassette has a transcriptional terminator within the tbb-2 3’UTR, we reason that any downstream regulatory sequences would likely have minimal impact on transcription, if any. This is different from said scenarios, such as normal N- or C- terminal gfp tagging without additional transcriptional terminator inserted.

Page 18: “Because the inserted cGAL gene trap cassette contains a transcriptional terminator in the tbb-2 3’ UTR, depending on the insertion sites—for example, those with relatively large introns of the targeted gene after the insertion site—the cGAL gene trap may not fully capture all the regulatory elements for the expression of the target gene.”

Minor suggestions:

1. p27. Figure 2, panel C. It would be useful to indicate the nature of the "tanSi67" transgene in the figure, even though the legend explains why these experiments should be rescued.

We agree with the reviewer that this can be helpful to effectively convey the message. We keep the original version, because the change will make the x-axis label become very long and create a large white space of the entire figure 2. Nonetheless, we did make the suggested change in the main text (see the next comment).

2. Line 84: Please add the allele name of the single-copy insertion (tanSi67)

We have added the allele name as suggested.

Page 9: “We found that the expulsion defects are fully rescued by crossing aex-2(tan75) mutants to tanSi67, a single-copy 11xUAS::aex-2(+) cDNA::linker::gfp::tbb-2 3’UTR effector line (Fig 2C).”

Reviewer #3:

Major points:

1. It would be very helpful to the C. elegans field and any labs who would like to use this system if the authors can elaborate on the rationale behind the choice of introns or positions within introns where the cassette was inserted. In particular, it would be useful to know if the authors have tested different introns/locations and if so whether the results are comparable. It is noted that in Figure 3, both tan174 and tan99 were examined in terms of the inx-16 loss-of-function phenotypes, however, it is unknown whether the cGAL driven expression of UAS::GFP are similar (only one of them is shown in Fig. 3F). Answers to this question will be important to understand the extent of variability in the expression of cGAL, considering the lack of clarity in the locations of regulatory elements for the targeted genes in C. elegans (despite the authors’ mentioning in Line 178 – 179 that “The cGAL driver is presumably expressed under the promoter of the targe gene”).

We thank this reviewer for this thoughtful comment. Based on the current results we have, both *inx-16(tan174)* and *inx-16(tan99)* alleles produced the same phenotype and the same expression pattern (specifically only in the intestine). We have added this new result in Fig 3G of the revised manuscript. In addition, we have added a paragraph in the Discussion section to discuss the rationale for insertion site selection.

Fig 3F and 3G, both inx-16 gene trap lines showed expression specifically in the intestine, when combined with 11xUAS::GFP effector.

Pages 16-17: “Considerations for the insertion site of the cGAL gene trap cassette

In our study, we tested our cGAL gene trap design by inserting the cGAL gene trap cassette in introns of the target genes and our results showed that gene trap works robustly for all the genes tested. For example, both inx-16(tan99) and inx-16(tan174), with the cGAL gene trap cassette inserted at different introns, showed the same mutant phenotype and expression pattern (Fig 3). We recommend considering the following factors when selecting an insertion site to generate a new gene trap for the gene of interest: 1) select an intron of your target gene with a specific guide RNA sequence (http://genome.sfu.ca/crispr/search.html, [30]) to insert the gene trap cassette into the genome via CRISPR; 2) if the main goal is to use the gene trap as a “promoter trap” by only focusing on the enhancer and regulatory elements in the promoter, then choose an insertion site within an intron closer to the start of the gene; 3) if the main goal is to use the gene trap to capture as many regulatory elements within the introns of the gene in addition to the promoter, then choose an insertion site within an intron closer to the end of the gene; 4) if the main goal is to use gene trap as a loss-of-function mutant, then choose an insertion site upstream of the coding sequence for critical domains of the target gene. Furthermore, as the cGAL gene trap cassette contains an added splice acceptor followed by multiple stop codons in all three reading frames, in principle, the gene trap strategy should also work even if the cassette is inserted into exons of target genes.”

2. In Figure 4C, the expression pattern of most of the bipartite systems are similar to the GFP pattern in Fig. 3E, except LexA GT, where the prominent GFP signals in head GABAergic neurons are missing. Is the image representative? If so, the authors should provide interpretation or comments and consider modifying the text about all of them being the same as Fig. 3E.

Thank you for pointing this out. We re-examined our images of LexA GT and observed GABAergic neurons in the head region. For unknown reasons, the GFP signal in GABAergic neurons in the head region is much weaker than the signal in those neurons along the ventral cord, which makes it a bit difficult to see those head neurons. We have selected a different image for LexA GT to better show the GABAergic neurons in the head region in Fig 4C. We have modified the texts in the revised manuscript.

Page 15: “When combined with their corresponding GFP effector lines, these new gene traps also drive GFP expression in the GABAergic neurons (Fig 4C), largely similar to the unc-47 cGAL gene trap (Fig 3E), suggesting these gene trap lines all reflect the endogenous expression pattern of unc-47. For reasons unknown, GFP signal in the GABAergic neurons in the head region of the unc-47 LexA gene trap appeared to be much dimmer than the signal in those neurons along the ventral cord.”

3. in Figure 2D, one more negative control where only syIs371/+ and Histamine are present would be required. More than one variable is changed between current groups.

Thank you for pointing this out. We are sorry that we did not explain the negative control very well in the original manuscript. The *aex-2(sa3)/+; syIs371/+* with histamine is the negative control, as it also contains one allele of aex-2(+) and one allele of aex-2(lf) allele, like the experimental group (aex-2(cGAL GT)/+; syIs371/+). Nonetheless, we also included the suggested negative control of syIs371/+ with histamine, which showed normal expulsion. The new data is included in Fig 2D (the rightmost column, see below).

Pages 11-12: “As negative controls, *aex-2(sa3)/+; 15xUAS::HisCl1/+* double heterozygous animals and *15xUAS::HisCl1*/+ animals showed normal expulsion even in the presence of 10 mM histamine (Fig 2D).”

Minor points:

1. For all bipartite-driven expression pattern data, negative control lacking the driver should be shown (e.g. Fig. 2 GH, Fig. 3EF, Fig. 4C).

We have added the negative control of the UAS effector line in the supplemental figure Fig S5 and made changes to texts in the revised manuscript.

Page 10: “When crossing aex-2(tan75) with a single-copy UAS::fluorescent protein effector line, we detected fluorescence signals in head and tail neurons, enteric muscles, and head mesodermal cell (HMC) (Fig 2G, 2H, and S4 Fig), which were not observed in animals with the UAS::fluorescent protein effector line alone (S5 Fig).”

Those effector lines for other bipartite expression systems in Fig 4C have been characterized previously and have been shown to have minimal background expression, if any (Nonet, Genetics, 2020). We have added the following sentence with the reference paper to the revised manuscript.

Page 15. “The different effector strains we used for the new unc-47 gene trap lines are from a previous study and they alone have minimal background expression, if any [11].”

2. In Figure S4, the choice of words “GFP (optimized for..)” is misleading. One option is to say “GFP intensity scaling optimized for…”.

We agree with this reviewer and have made the change as suggested.
